# Gaps in research and capacity development for malaria surveillance and response in the Asia–Pacific: meeting report

**DOI:** 10.1186/s12936-023-04459-9

**Published:** 2023-03-10

**Authors:** Massaya Sirimatayanant, Phone Si Hein, Laura Fay Anderson, Lucía Fernández Montoya, Rebecca Potter, Mwalenga Nghipumbwa, Prasad Ranaweera, Pengby Ngor, Rattanaxay Phetsouvanh, Richard J. Maude

**Affiliations:** 1 Mahidol-Oxford Tropical Medicine Research Unit, Faculty of Tropical Medicine, Mahidol University, Bangkok, Thailand; 2 Asia Pacific Malaria Elimination Network (APMEN), Singapore, Singapore; 3 Global Malaria Programme, World Health Organization, Geneva, Switzerland; 4 University of Oslo, Oslo, Norway; 5 Anti-Malaria Campaign, Ministry of Health, Colombo, Sri Lanka; 6 National Centre for Parasitology, Entomology and Malaria Control, Phnom Penh, Cambodia; 7 Department of Communicable Disease Control, Ministry of Health, Vientiane, Lao PDR; 8 Centre for Tropical Medicine and Global Health, Nuffield Department of Medicine, University of Oxford, Oxford, UK; 9 Harvard TH Chan School of Public Health, Harvard University, Boston, USA; 10 The Open University, Milton Keynes, UK

## Abstract

**Background:**

This report is based on the 2021 annual meeting of the Asia–Pacific Malaria Elimination Network Surveillance and Response Working Group held online on November 1–3, 2021. In light of the 2030 regional malaria elimination goal, there is an urgency for Asia–Pacific countries to accelerate progress towards national elimination and prevent re-establishment. The Asia Pacific Malaria Elimination Network (APMEN) Surveillance Response Working Group (SRWG) supports elimination goals of national malaria control programmes (NMCPs) by expanding the knowledge base, guiding the region-specific operational research agenda and addressing evidence gaps to improve surveillance and response activities.

**Methods:**

An online annual meeting was hosted from 1 to 3 November 2021, to reflect on research needed to support malaria elimination in the region, challenges with malaria data quality and integration, current surveillance-related technical tools, and training needs of NMCPs to support surveillance and response activities. Facilitator-led breakout groups were held during meeting sessions to encourage discussion and share experience. A list of identified research priorities was voted on by attendees and non-attending NMCP APMEN contacts.

**Findings:**

127 participants from 13 country partners and 44 partner institutions attended the meeting, identifying strategies to address malaria transmission amongst mobile and migrant populations as the top research priority, followed by cost effective surveillance strategies in low resource settings, and integration of malaria surveillance into broader health systems. Key challenges, solutions and best practices for improving data quality and integrating epidemiology and entomology data were identified, including technical solutions to improve surveillance activities, guiding priority themes for hosting informative webinars, training workshops and technical support initiatives. Inter-regional partnerships and SRWG-led training plans were developed in consultation with members to be launched from 2022 onwards.

**Conclusion:**

The 2021 SRWG annual meeting provided an opportunity for regional stakeholders, both NMCPs and APMEN partner institutions, to highlight remaining challenges and barriers and identify research priorities pertaining to surveillance and response in the region, and advocate for strengthening capacity through training and supportive partnerships.

**Supplementary Information:**

The online version contains supplementary material available at 10.1186/s12936-023-04459-9.

## Background

Between 2000 and 2020, estimated malaria incidence and mortality reduced significantly in the WHO South-East Asia region (SEARO) by 83% and 81%, respectively. The region met the Global Technical Strategy (GTS) 2020 milestone of reducing mortality and morbidity by 40% from a 2015 baseline [1]. However, in the Western Pacific region (WPRO) there was an overall increase in incidence and mortality between 2015 and 2020 and malaria is expected to persist until 2030 [1]. In light of the 2030 regional elimination goal, there is increasing urgency for Asia–Pacific countries to accelerate progress towards national malaria elimination, concentrating on high burden geographies, and prevent re-establishment [2].

The Asia Pacific Malaria Elimination Network (APMEN) was established in 2009 and has since grown to host a forum of 21 country partner National Malaria Control Programmes (NMCPs) committed to eliminating malaria within their borders and supporting region wide elimination by 2030. Under APMEN, three working groups provide technical support and guidance on key themes of malaria control and management, namely, vector control, surveillance and response, and *Plasmodium vivax.* Through a network of partner institutions including non-governmental organizations, academic and research institutions, funding agencies, the private sector, United Nations agencies, and civil society organizations, APMEN provides country partners with access to technical knowledge, tools, and expertise to support their respective national and regional elimination goals. The Asia Pacific Leaders Malaria Alliance (APLMA) and APMEN Secretariats were brought together in 2015 to strengthen elimination efforts by combining the political advocacy and multi-sectoral access of APLMA with APMEN’s technical expertise and direct engagement with malaria control programmes [3].

With the transformation of malaria surveillance as a core intervention under the WHO Global Technical Strategy (GTS) [4], the APMEN SRWG has been supporting expansion of the knowledge base, development of a region-specific operational research agenda and addressing evidence gaps to ensure that country partners can effectively implement surveillance strategies that identify and respond to every malaria case [5]. As a peer-led technical working group, the hosting arrangement of the SRWG rotates biannually and is currently co-chaired by Dr Rattanaxay Phetsouvanh, Director General of the Department of Communicable Disease Control, Ministry of Health in Lao PDR, and Professor Richard J. Maude, Head of the Epidemiology Department at Mahidol Oxford Tropical Medicine Research Unit (MORU) for 2021–2022.

Annually, the SRWG hosts a general membership meeting bringing together country partners and experts from across the world to reflect on recent developments and address key challenges pertaining to malaria surveillance activities in the region. In line with the 2021 updated strategy highlighting the need to integrate malaria services into broader health delivery systems and strengthen countries’ capacity to collect, analyse and use malaria-related data [6], the SRWG focused this year’s annual meeting around the theme of “Moving from Data to Elimination” through building capacity in research, data quality, and integration. Due to the COVID-19 pandemic, the 2021 meeting was organized virtually on 1–3 November and was attended by 127 participants comprising 13 country partner NMCPs, and 44 partner institutions from 24 countries across the world (the meeting agenda and list of attending organizations is found in Additional Files 1, 2, respectively). This meeting report documents outcomes from the virtual meeting, key discussion points from the breakout sessions and findings from surveys conducted as part of the proceedings.

### Data to elimination: prioritizing gaps in research and capacity development for surveillance and response

The WHO GTS outlines a key supporting element to achieving elimination requires leveraging innovation and expanding both clinical and implementation research [4]. The last SRWG annual meeting organized in 2018 discussed challenges and barriers to identifying and implementing solutions for improving case-based surveillance and response activities and, among other goals, to review approaches and tools for improving surveillance activities [7]. The 2021 SRWG annual meeting provided an update on partner perspectives regarding remaining challenges and barriers to implementing surveillance and response activities, what research is needed to address challenges to eliminating malaria in the region, review current surveillance-related technical tools, and reflect on training needs of national programmes to support surveillance and response activities. A summary of the session goals, outcomes and action points can be found in Table 1.

**Table 1:** Meeting output and action points

Session goals	Session outputs	Action points
Session 1: building capacity through research		
Discuss, identify and prioritize what research is needed to support malaria elimination in the Asia–Pacific region	- List of research questions submitted by NMCPs in different sub-regions and APMEN partner institutions (Additional File 1) - **Priority research in order of votes:** 1. Strategies to address malaria transmission amongst mobile and migrant populations (MMPs) 2. Cost effective surveillance strategies to maximize in low resource setting 3. Integration of malaria surveillance with the broader health system 4. Minimal surveillance package for monitoring and evaluation for sustaining malaria free status	- **Build capacity for elimination through research by:** 1. Openly and widely disseminating identified research priorities 2. Providing linkage with APMEN research institutions to conduct the research needed in specific countries 3. Advocating to potential donors to fund the identified research priorities
Session 2 & 3: building capacity through data quality, integration and technology		
Identify key challenges to, and solutions for, improving data quality Identify key challenges to, and solutions for, integrating epidemiology and entomology data for malaria surveillance Review technical solutions for malaria elimination	- **Data quality challenges:** 1. Limited human resources and technological capacity for data collection and entry processes 2. Difficulty in tracing mobile and migrant populations 3. Poor or lack of Standard Operating Procedures (SOP) and monitoring and evaluation of both data collection teams and data quality 4. Competing priorities for data collectors 5. Complex reporting and poor data management systems - **Data quality** **solutions:** 1. Clear SOPs for case investigation 2. Simplified reporting systems and digitization of data collection tools 3. Improved monitoring and supervision of data collection units 4. Establishment of a national quality assurance system for malaria diagnosis 5. Routine data cleaning and review 6. Integration of private sector data and supplementing with geospatial information - **Data integration challenges:** 1. Divergence in spatial resolution, coverage and frequency of data collected 2. Costliness and lack of capacity to collect entomological data 3. Extrapolating vector surveillance data geographically 4. Lack of data management systems supporting integration of both types of data - **Data integration solutions:** 1. Correlating data collected at the same geographical location and time 2. Coordinating data collection between epidemiological and entomological data collection teams 3. Unifying data collection and management systems - **Technical solutions:** 1. WHO’s Digital Solutions for Malaria Elimination initiative supports the use of digital tools to strengthen an integrated surveillance information system on the DHIS2 platform 2. Utilization of malaria packages under DHIS2 metadata tools to leverage the health management information system 3. Google data studio as complementary digital dashboard for malaria case-based and drug stock surveillance in Sri Lanka 4. Nationally designed and tailored web-based Malaria Information System in Cambodia supports real-time reporting through mobile applications	- **Build capacity for elimination through improved data quality and integration by:** 1. Reviewing key challenges and barriers raised across national programs to target topics for implementing informative webinars and training workshops for the wider APMEN audience 2. Providing linkage with APMEN implementing partners to establish technical support initiatives
Session 4: moving from data to elimination		
Identify ways to strengthen capacity to utilize data for action	- Inter-regional partnerships with the Roll Back Malaria Surveillance, Monitoring and Evaluation Reference Group’s Surveillance Practice and Data Quality (SMERG SP&DQ) Committee - **Training topics identified from training needs assessment and breakout group discussions:** 1. Malaria vector surveillance 2. Case and foci investigation 3. GIS and mapping 4. Data quality assurance in the surveillance data pipeline	- **Build capacity for elimination through training by:** 1. Developing cross-regional training workshops 2. Designing and delivering training workshops for data quality dimensions and GIS and mapping 3. Establishing technical support initiatives to improve the quality of case and foci investigation in specific countries that have expressed interest

### Research needed to support malaria elimination in the Asia–Pacific region

Breakout groups dividing participants by subregion and APMEN membership type allowed identification of research priorities stratified by sub-regional needs and organizational focus. Facilitators asked participants to submit research questions on Slido, an interactive application used for hybrid meetings, clarify components of the research question, and vote on the priority questions from the full list of research questions submitted within each subgroup. Research questions from all subgroups were pooled and grouped by themes, and opened for voting by all participants of the annual meeting on the Qualtrics XM platform, to derive a region-wide list of ranked research priorities. Follow-up email invitations to participate in the survey were distributed to contacts from APMEN country partners not present at the meeting (18 countries, including 7 not at the meeting). In total, the survey received 14 responses from eight country partners, and 27 responses from partner institutions deriving the list of top-ranking research priorities. The full list of research questions identified by each subgroup and ranked by priority is in Additional File 1.

Strategies for malaria elimination to address malaria transmission amongst mobile and migrant populations (MMPs), including forest goers, was the highest voted research priority by both country partners and partner institutions. Transmission of malaria in near elimination settings, particularly the Greater Mekong Subregion (GMS), often happens in forests and in cross-border areas where human mobility is high [8]. Because of this, village-based malaria control and elimination strategies common in the GMS cannot provide effective monitoring, health education or care service coverage for this vulnerable high-risk group [9, 10], therefore, they may act as reservoirs of malaria [8]. Research to determine healthcare-seeking behaviours and mobility patterns of MMPs and those populations living at the borders where transmission is harder to trace, will be essential to monitoring high risk-groups and disease transmission patterns. Break-out group participants discussed how mobile and migrant population movement mapping, within and across national borders, will be vital research to inform surveillance and response strategies for targeting the last remaining transmission foci and support elimination in many Asia–Pacific countries.

The second top voted research question was determining cost effective surveillance strategies to maximize efficiency in low resource settings. To achieve elimination, the 2017 World Health Organization (WHO) Framework for Elimination [11] suggested that surveillance systems transition from reporting aggregate case data towards case-based surveillance to rapidly identify, investigate, classify, report and respond to all individual malaria cases to effectively manage cases and implement informed vector control interventions. An effective surveillance and response system that detects and responds to every new case and focus can therefore be resource intensive [12]. Many malaria programmes in low resource settings rely on donor or international funding which is often influenced by changing international public health landscapes and priorities that subsequently impact the consistency and sustainability of financial assistance [13, 14]. Contextualized surveillance strategies that are cost effective will be a priority for many malaria programmes in the region that have to maximize use of declining resources to maintain momentum for elimination or sustain efforts against re-establishment in the post elimination phase.

Other top research priorities identified by country partners included integration of malaria surveillance with the broader health system, and minimal surveillance packages for monitoring and evaluation to sustain malaria free status. While guidelines for both research priorities exist in the WHO Malaria Surveillance, Monitoring and Evaluation Reference Manual [12], operationalization remains challenging for national programmes [15]. Research is needed to identify the best tools and practices for how to operationally implement adaptive surveillance systems that integrates malaria services and indicators into the general health service and information systems, and standardize tools for monitoring the quality of malaria surveillance in low to zero transmission settings [15].

### Challenges and solutions to ensuring high quality surveillance data to guide decision making

Dr Laura Fay Anderson (Global Malaria Programme—GMP, WHO) presented the WHO’s Malaria Surveillance Assessment Toolkit [16] as an introduction to the session on surveillance and data quality. The toolkit includes a minimum set of priority indicators that allow comparable and replicable malaria surveillance assessments across multiple countries or within the same country over time, and is adaptable to country context by allowing users to define the assessment scope. For eliminating countries with very low levels of transmission, the toolkit can be used to evaluate a surveillance system’s ability to capture and respond to every case and show preparedness to prevent re-establishment. For all levels of transmission, the assessment allows for evidence-based and prioritized recommendations to strengthen surveillance systems and ensure that response activities are well targeted. Currently, the WHO is piloting the toolkit to carry out comprehensive assessments in five African countries. As this effort had not expanded to the Asia–Pacific, randomly allocated breakout groups were created in the session that followed to discuss challenges and solutions to ensuring high quality surveillance data in this region.

Participants across breakout groups cited challenges in the data collection processes that impact surveillance data quality, ranging from issues with collecting data using mobile populations, within and across countries, to limitations pertaining to data collection procedures. Participants indicated difficulty in tracing MMPs leading to double counting and losses from follow-up across subnational and international borders. Participants also noted that poor or lack of standard operating procedures (SOPs) for field data collection, and diverging quality of case information and definitions impact data completeness and accuracy. Limited human resources and capacity for data collection and entry due to insufficient training, difficulty in reaching remote areas, and high workload for staff compiling and entering data also affected the timeliness of data availability and monthly reporting. Lack of monitoring and evaluation processes, including SOPs for checking data quality, were also noted as a challenge.

Clear SOPs and guidelines on when and how cases are investigated, and use of simplified forms and user-friendly reporting systems were suggested as possible solutions. Participants also proposed staff monitoring and supervision through data entry feedback sessions and establishing data collection groups to review data. Good training tools and methods to motivate staff, such as by stressing the importance of surveillance data for policy responses, are other ways to improve human resources and capacity for data collection and entry when there are competing priorities. Highlighting high achieving data collection units was suggested as a way to motivate staff members. Finally, as countries move towards elimination, a national quality assurance system for malaria diagnosis was indicated as vital for ensuring malaria surveillance data quality.

To ensure completeness of data, simple routine data cleaning and checking involving following up on missing and incomplete data was suggested. Involvement of the private sector, a major provider of malaria diagnosis and treatment in many countries [17], in the malaria surveillance system will also be vital to capture the whole picture of malaria endemicity.

Limitations cited related to tools or technologies, including issues with internet connectivity and limited use of modern technology to collect and upload data in remote areas. Digitalization of data through the use of tablets or mobile phones for data collection and developing localized data entry systems were discussed as possible solutions. Additionally, participants explained that complex data collection tools, with multiple forms and reporting systems used in many countries, may lead to duplication and inconsistent data especially when comparing surveillance data collected by public and private health facilities. Inadequate storage and management of data were indicated as data quality challenges, citing difficulties in integrating multiple data streams and catchments as well as data platforms used for different types of data and/or funding sources. Designing contextualized digital data dashboards which integrate multiple data streams will be key to addressing this challenge and promoting data utilization.

Participants described how suboptimal data quality impacts decision making for planning and targeting interventions, and resource management. Incorrect case classifications, non-standardization of case definitions that lead to varying interpretation across programmes, and gaps in knowledge about specific surveillance elements such as entomological, geographical or demographic were all identified to negatively impact data used to inform intervention planning and decision making by NMCPs. When data is not contextualized accurately in space and time, resources may not be efficiently prioritized and programmes may not be able to respond to the needs around RDT and drug stocks, and training. Waste of resources further reduces programme cost effectiveness. Data delays hamper ability to provide timely interventions to respond to cases and outbreaks.

### Challenges and solutions to integrating epidemiological and entomological data for decision making

In her presentation, Dr Lucía Fernández Montoya (GMP, WHO) highlighted the need to integrate routine entomological surveillance data with epidemiological and environmental information to provide a holistic understanding of malaria transmission dynamics and guide malaria control efforts. Malaria is transmitted by *Anopheles* mosquito vectors and vector control intervention are among the most effective intervention for malaria control [18]. Each vector control intervention targets different characteristics of mosquito vectors, for example, IRS targets indoor resting adult vectors, ITNs target indoor biting adult vectors, larviciding targets immature vector stages. Different *Anopheles* species have varying preferences for environmental conditions (e.g. forest, forest fringe, rice field); resting and biting location (indoor/outdoor), biting times and feeding (humans or animal blood) which determine both their vulnerability to each vector control intervention and malaria transmission patterns [12, 19].

On its own, entomological surveillance can help to identify the main vectors that should be targetted and identify challenges to effective vector control (e.g. insecticide resistance, vector exophily or exophagy, and changes in vector composition). Combined with epidemiological and other types of data, it can help to determine receptivity and vulnerability to malaria transmission in different areas; select appropriate vector control interventions and their deployment modalities; monitor and evaluate the effectiveness of vector control interventions; investigate the causes of malaria outbreaks, unexpected patterns of transmission and drivers of transmission in transmission foci; and evaluate the risk of reintroduction of malaria into areas where it was previously eliminated [12].

Entomological surveillance can be conducted using different vector collection methods, each of which provides different types of information and allows for the calculation of only a subset of core entomological indicators (19). Entomological surveillance systems and derived indicators must therefore be carefully designed to provide useful information for programme planning and implementation [12]. The timing and location of entomological data collection should also be planned to address programme information needs.

Participants were randomly allocated into breakout groups to identify challenges to integrating epidemiological and entomological data. The first major challenge identified was differences in the frequency and geographical scale of data making combined analyses difficult. Entomological data are generally collected during foci investigation or surveys in relatively small areas for specific time periods, whereas epidemiological data is collected continuously from every heath provider/facility. Another challenge is the difficulty and high cost of collecting entomological data, especially in deep forested areas where vulnerability to malaria transmission is generally high. Entomological data collection also requires specific equipment, that needs to be transported to the field and frequently replaced, and specialized training to use it.

Challenges to analysing entomological data due to its complexity, and with extrapolating vector surveillance data across all areas, were also discussed by participants. These lead to limited utilization of entomological data in designing interventions and responses. Participants further identified lack of data sharing between entomology and epidemiology teams, and lack of feedback on reported data across the program and implementers as a challenge to data use and integration. Participants also identified differences in the systems used for the collection of epidemiological and entomological data as a challenge for data integration. In some countries, epidemiological data is collected through DHIS2, while a different system is used for collecting entomological data. In some cases, several different systems are used for the collection of entomological data within the same country, which further hampers data integration.

Integration will require determining the optimal spatial and temporal granularity of different data so they can be combined to inform decision making. Ways to enhance the resolution of entomological data to better match epidemiological data scales were discussed, including by conducting vector suitability mapping using satellite data. Data may be integrated by visualizing entomological and epidemiological data together to explore patterns and trends between the two types of data. Coordination between epidemiological and entomological data collection teams, and between national programmes and partners, is also essential for successful data integration. Finally, using the same data management platform for both data was felt to be important for proper integration.

### Technical solutions for malaria elimination

The WHO outlines a key supporting element to achieving elimination requires leveraging innovation in addition to expanding both clinical and implementation research [6]. New technologies, such as mobile applications and digital platforms, can improve surveillance activities and quality of surveillance data. Such technologies can speed up data collection, reporting, consolidation, feedback, sharing and presentation. Moreover, information technology can enhance procurement and supply chain, service delivery, and financial and other resource mobilization. The final session of the annual meeting covered various technical solutions to improve malaria surveillance.

An optimal, fully integrated malaria information system should include the collection of complete and correct data, real-time reporting, storage and integration, data analysis and visualization, data sharing and active and continuous data interpretation. Rebecca Potter (University of Oslo) presented the DHIS2 malaria toolkit which was developed in partnership with WHO GMP to reflect global normative guidance and WHO recommendations. The toolkit includes standardized metadata packages for case notification, investigation, and classification workflows, as well as foci investigation and response. The packages are designed to make it easier for NMCPs to adopt global recommendations for malaria data collection and analysis into their national information systems that are using DHSI2 software. Data-quality tools and pre-configured dashboards for malaria surveillance in elimination settings support NMCPs to implement rapid data-driven responses. Collaboration with GMP was planned to expand the DHIS2 toolkit for supporting integration of entomological data. These tools were developed to facilitate the integration of malaria data into the broader national health information system.

The importance of the quality, availability, and utilization of malaria surveillance data for decision-making to better tailor and target programmatic activities is highlighted in the WHO GTS for Malaria [6]. This has led to countries adopting online or electronic malaria systems to notify cases within 24–48 hours, support timely reporting, and implement real-time case and foci surveillance and vector mapping. Mwalenga Nghipumbwa (GMP, WHO) presented on how the WHO is supporting the enhancement and development of existing and new digital tools to strengthen these integrated near-real time surveillance systems. The digital solutions for malaria elimination (DSME) community developed effective mobile application tools connected to upgraded core DHIS2 functionalities to simplify complete, timely and accurate data reporting. The suite of tools include the DHIS2 Android Capture app [20] and OpenSRP platform [21] used to support real-time reporting and case notification, as well as foci investigation.

NMCP applications of various digital solutions to support malaria surveillance systems were presented for Sri Lanka and Cambodia. Dr. Prasad Ranaweera (Ministry of Health and Indigenous Medicine, Sri Lanka) demonstrated the use of Google Data Studio to easily upload data and customize dashboards for tracking antimalarial drug stocks and RDTs. The use of this platform complements the DHIS2 system, and was introduced to the Anti-Malaria Campaign in Sri Lanka during the post elimination phase. Dr Pengby Ngor (MORU/National Center for Parasitology, Entomology and Malaria Control) described Cambodia’s standalone malaria information system (MIS), which captures both passive and active surveillance data in real-time through health centres and village-based malaria workers, respectively, using a web-based interface and Android smartphone malaria surveillance applications. While challenges remain with device maintenance and providing regular refresher training for turnover of staff, the locally designed application is low-cost, sustainable, decentralized and tailored to the country’s malaria elimination context. Plans are being made to adapt this tool for the surveillance of other diseases.

### Capacity and training needed for national malaria control programmes

The final session focused on sharing cross-regional initiatives on accruing surveillance best practices and evaluating the barriers to, and gaps in, training for country partners needed to strengthen surveillance and response activities. Dr Arantxa Roca-Feltrer (Malaria Consortium), co-chair of the Roll Back Malaria Surveillance Monitoring and Evaluation Reference Group (RBM-SMERG) introduced upcoming initiatives and resources under the committee for Surveillance Practice and Data Quality (SP&DQ), including development of a systematic tracker of implementing partners’ data quality projects, and NMCPs’ use of surveillance practices. Anne-Sophie Stratil (Malaria Consortium) presented findings from a needs assessment of NMCPs in African countries, identifying priority challenges in using data for program and policy decisions, assessing data quality, reporting, collecting, visualizing and interpreting program data. While the RBM-SMERG has traditionally been an African centric group, collaborations and exchanges with the APMEN SRWG are planned to support exposure and interaction across regional bodies to capture activities and best practices from the full spectrum of endemic countries in the two regions.

Findings from a survey of APMEN partner countries’ training needs and partner institutions’ training capacity conducted in 2021 were presented by Massaya Sirimatayanant (MORU/SRWG). The survey received 40 responses (22 from country partners and 18 from partner institutions) identifying country partners’ interest in skills-based training. The survey also identified APMEN partner institutions with ability and interest to provide training in topics of interest to country partners. Virtual face-to-face follow-up discussions clarified the specifics of the training contents and target audiences. From this, five priority training topics were identified as of most interest to NMCPs: entomological surveillance, data utilization (data to action), basic statistical analysis, mapping and GIS, and case investigation. Feasibility to deliver, and interest in receiving, training on these topics were further explored in self-assigned breakout group sessions.

For entomological surveillance, shortfalls and training needs in the Asia Pacific have been identified and prioritized across sub-regions by the APMEN Vector Control Working Group (VCWG). Upcoming VCWG initiatives including a 6-module online training on malaria vector surveillance and a face-to-face two-week intensive vector surveillance course were discussed for 2022.

In the breakout group discussing case and foci investigation, participants acknowledged that training is already being conducted by NMCPs in countries. A framework for defining cases exists and may be used to deliver basic training across countries, but participants noted that training for classification of cases and transmission sites will be unique to each country context and require training that is adapted to each transmission setting. Participants discussed the potential to deliver training to improve the quality of case and foci investigation through bilateral NMCP-NMCP or NMCP-partner institution technical support initiatives facilitated by the SRWG.

In the GIS and mapping breakout group, a survey found that a mix of NMCP staff at national and sub-national levels had already received GIS/mapping-related training. There was interest to strengthen capacity for central level staff through introductory and more advanced training to use geospatial software for malaria surveillance and monitoring, and at sub-provincial levels training should be focused on introducing geospatial data and technologies for surveillance and how to collect geographic coordinates in the field. An online training module on GIS for mapping infectious diseases is under development by Dr Steeve Ebener (Health GeoLab Group in MORU Epidemiology Department) and Prof Richard Maude (MORU), and online workshops adapted for malaria are expected to be delivered via APMEN in 2022.

Finally, for the combined training themes of data utilization and basic statistical analysis, participants expressed a preference to focus on data processes, including data collection and quality checking, rather than basic statistical analyses. This focus was acknowledged by the SRWG, and training on quality assurance within the surveillance data pipeline will be a priority deliverable in 2022.

## Discussion and next steps

While there has been steady progress towards achieving the goal of regional elimination by 2030, a number of research gaps, implementation challenges and training needs pertaining to surveillance and response activities have been identified during the SRWG annual meeting for Asia–Pacific countries. Action points resulting from the meeting outcomes are outlined in Table 1.

The higher active attendance of stakeholders from the GMS region (44 percent of total attendees across all three days) may have contributed to identification of research priorities that reflect challenges currently prominent in low transmission settings. However sub-regional level breakout group discussions and voting on research priorities that were open to the wider regional audience allowed identification of other top research priorities, including cost effective surveillance strategies and integration of malaria surveillance into broader health systems, which are highly relevant for countries in all transmission settings in the region. Globally, funding for malaria has remained stagnant since 2010 [22], and with competing domestic public health priorities, ensuring that malaria interventions and surveillance strategies are cost effective and well-integrated into the wider public health system will be vital for sustainability of malaria programmes. Identification of key research priorities needed to support malaria elimination according to stakeholders will be critical to guiding operational research at the sub-regional level and across the Asia Pacific. Their dissemination to a wider audience to guide linkages between research institutions and NMCPs, and to potential donors, will be a target action point for the SRWG to support ongoing elimination efforts.

Discussions surrounding surveillance data quality and integration challenges, particularly the limited examples of initiatives targeting improvement in these within the region, suggests that there are significant gaps in research and best practices for both themes. While there have been some piloted efforts in the GMS to improve completeness of malaria incidence data, such as through integration of private sector data [17, 23], a past assessment of national surveillance systems in the Asia Pacific found malaria incidence data in many countries still misses information from a wide range of potential sources [24]. Furthermore, while pilots of digital tools for improving timeliness of malaria case data collection have been explored [25–28], difficulty with scaling and challenges in reporting timeliness as discussed in the breakout group highlight its persistent impact on NMCP’s ability to implement targeted and timely responses to outbreaks. While the GTS has emphasized the importance of high-quality routine data by redefining surveillance as a core intervention in malaria control and elimination[4], there have been limited efforts to evaluate programmes’ ability to capture quality routine malaria surveillance data when compared to Africa [29–33]. High quality of routine surveillance data and integration of various sources and types of data are needed to provide a complete picture of malaria incidence within a country in order to successfully plan for malaria control and elimination and inform targeted response strategies. Advocacy to prioritize data quality assurance measures will be sought through hosting APMEN-supported informative webinars and workshops. Due to the unique nature of different surveillance systems and Health Management Information Systems (HMIS) adopted across countries in the region, the SRWG will seek to provide context specific technical support by linking NMCPs with partner institutions with the relevant expertise.

Group brainstorming sessions complemented a previously implemented training needs assessment by offering the wider SRWG membership to clarify training content that partner countries are interested in receiving. In comparison to the pre-determined training topics list provided for respondents to select in the training needs assessment survey, the breakout group facilitated discussions between NMCPs and partner institution members to suggest deviations from the original topics based on contextual needs and regional expertise available. Both activities determined which training may be delivered to a wider APMEN audience (vector surveillance and GIS and mapping), and which require context specific technical support (case and foci investigation). Engaging RBM’s Committee on Surveillance Practice and Data Quality, active in the African content, allows the SRWG to co-develop cross-regional training workshops on surveillance data quality that are of relevance to both regions.

## Conclusion

While progress towards elimination has been made across the region, the global COVID-19 pandemic has tested the resilience of national malaria programmes and their ability to implement surveillance and response activities for malaria amidst lockdowns and competing public health priorities. The 2021 SRWG annual meeting provided an opportunity for regional stakeholders, both NMCPs and partner institutions, to highlight the remaining challenges and barriers, update on and share innovative technologies, and advocate for both region-wide as well as context specific initiatives to strengthen surveillance and response activities. Identification of research and NMCP training priorities are vital to ensuring that supportive initiatives within the region, including SRWG-led activities, are well-targeted to meet the needs of countries for building capacity to achieve elimination by 2030.

## Supplementary Information

**Additional file 1: **Meeting agenda.

**Additional file 2: **List of attending country partner National Malaria Control Programmes and partner institutions.

**Additional file 3: **Breakout groups and identified research priorities on malaria surveillance in the region.

## Data Availability

Data sharing not applicable as no datasets were generated or analysed in this meeting report.

## Abbreviations

SEARO: South-East Asia region (SEARO)
WPRO: Western Pacific region
APMEN: Asia Pacific Malaria Elimination Network
SRWG: APMEN Surveillance and Response Working Group
NMCP: National malaria control programme
APLMA: Asia Pacific Leaders Malaria Alliance
GTS: Global technical strategy
MORU: Mahidol Oxford tropical medicine research unit
MMP: Mobile and migrant populations
GMP: Global malaria programme
DHIS2: District health information software
SOPs: Standard operating procedures
GMS: Greater Mekong subregion
DSME: Digital solutions for malaria elimination
MIS: Malaria information system
RBM-SMERG: RBM surveillance monitoring and evaluation reference group
SP&DQ: Surveillance practice and data quality committee
VCWG: APMEN vector control working group
HMIS: Health management information system
