# Supplementary material for: Gaps in research and capacity development for malaria surveillance and response in the Asia–Pacific: meeting report
Source: Malar J. 2023 Mar 10;22:91. doi: 10.1186/s12936-023-04459-9 (PMC10000341; doi:10.1186/s12936-023-04459-9)
Supplement: Supplementary file 1 — Additional file 1: Meeting agenda. [file 12936_2023_4459_MOESM1_ESM.docx]

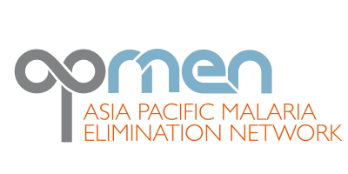


**APMEN Surveillance & Response Working Group Annual Meeting 2021**

**Agenda**

Theme: **Data to Elimination: Prioritizing gaps in research and capacity development for surveillance and response**

Date and time: **1 – 3 November 2021, starting at 13.00 hrs. (Singapore time)**

| **Start time** | **Durat-ion** | **Topic** | **Speaker** | **Organization** |
| --- | --- | --- | --- | --- |
| **Day 1 (1^st^ Nov 2021, Monday) Opening and Research priorities** | | | | |
| 13:00 | 00:05 | Opening | Dr Rattanaxay Phetsouvanh | MoH Lao PDR/ SRWG |
| 13:05 | 00:10 | Introduction of APMEN | Amita Chebbi | APLMA / APMEN |
| 13:15 | 00:10 | Introduction & agenda | Prof Richard Maude | MORU / SRWG |
| 13:25 | 00:05 | Group Photo |  |  |
| **Session 1: Building capacity through research** | | | | |
| 13:30 | 00:10 | Session introduction and groupwork overview | Session Chair  Dr Abdul Majeed | DoMC, Pakistan |
| 13:40 | 00:45 | Groupwork  *Prioritize research gaps with regards to malaria surveillance and response in the region* | 1. Dr Julia Dunn  2. Sazid Ibna Zaman  3. Dr Tanya Russel  4. Frank Kagoro  5. Dr Chris Chew  6. Rittika Datta  7. Wynn Zaw | 1. CHAI  2. MORU, Bangladesh  3. JCU  4. MORU/ WWARN  5. MORU, Bangkok  6. APLMA  7. MORU, Myanmar |
| 14:25 | 00:30 | Presentation & discussion | Session Chair / Facilitators |  |
| 14:55 | 00:05 | Closing | Session Chair |  |
|  | | | | |
| **Day 2 (2^nd^ Nov 2021, Tuesday) Building capacity through data quality and technology** | | | | |
| 13:00 | 00:05 | Synopsis of Day 1 & agenda for Day 2 | Massaya Sirimatayanant | MORU / SRWG |
| **Session 2: Data quality and integration for malaria surveillance** | | | | |
| 13:05 | 00:10 | Session introduction | Session Chair  Dr Siv Sovannaroth | CNM, Cambodia |
| 13:15 | 00:10 | WHO Surveillance Assessment Toolkit, with focus on the elimination module | Dr Laura Fay Anderson | GMP, WHO |
| 13:25 | 00:15 | Entomological surveillance for decision making | Dr Lucia Fernandez Montoya | GMP, WHO |
| 13:40 | 00:15 | Q&A | Session Chair |  |
| 13:55 | 00:05 | Groupwork overview | Session Chair |  |
| 14:00 | 00:35 | Groupwork  *1. Data quality*  *2. Data integration* | 1. Dr Chris Chew  2. Jade Rae  3. Dr Julia Dunn  4. Rittika Datta  5. Sazid Ibna Zaman  6. Wynn Zaw | 1. MORU Bangkok  2. METF, SMRU  3. CHAI  4. APLMA  5. MORU, Bangladesh  6. MORU, Myanmar |
| 14:35 | 00:20 | Presentation & discussion | Session Chair/ Facilitators |  |
| **Session 3: Technical solutions for malaria elimination** | | | | |
| 14:55 | 00:05 | Session introduction | Session Chair  Dr Siv Sovannaroth | CNM, Cambodia |
| 15:00 | 00:15 | Digital Solutions for Malaria Elimination (DSME) | Mwalenga Nghipumbwa | GMP, WHO |
| 15:15 | 00:10 | DHIS2 metadata packages and recent developments on malaria case-based surveillance and vector control packages | Rebecca Potter | University of Oslo |
| 15:25 | 00:10 | Digitalization of Anti Malaria Campaign Sri Lanka | Dr Prasad Ranaweera | Anti-Malaria Campaign, Sri Lanka |
| 15:35 | 00:10 | Smart phones for community health and MIS integration in Cambodia | Pengby Ngor | MORU / CNM, Cambodia |
| 15:45 | 00:15 | Q&A | Session Chair |  |
| 16:00 | 00:05 | Closing | Session Chair |  |
|  | | | | |
| **Day 3 (3^rd^ Nov 2021, Wednesday) Building capacity through training** | | | | |
| 13:00 | 00:05 | Synopsis of Day 2 and agenda for day 3 | Massaya Sirimatayanant | MORU / SRWG |
| **Session 4: Building capacity through training** | | | | |
| 13:05 | 00:05 | Session introduction | Session chair  Dr Luciano Tuseo | MME, WHO |
| 13:10 | 00:15 | Introducing the Surveillance Monitoring and Evaluation Reference Group, upcoming initiatives and training resources | Dr Arantxa Roca-Feltrer | SMERG, Malaria Consortium |
|  |  |  | Anne-Sophie Stratil | Malaria Consortium |
| 13:25 | 00:10 | Training Needs Assessment of APMEN SRWG | Massaya Sirimatayanant | MORU / SRWG |
| 13:35 | 00:10 | Q&A | Session chair |  |
| 13:45 | 00:10 | Group work overview | Session chair |  |
| 13:55 | 01:00 | Training Module Brainstorming Session: Clarification of modules for top training priorities  *1. Entomological surveillance (VCWG resources)*  *2. Data utilization / basic statistical analysis*  *3. Case and foci investigation*  *4. Mapping and GIS* | 1. Dr Leo Braack  2. Dr James Watson  3. Dr Chawarat Rotejanaprasert  4. Dr Steeve Ebener | 1. Malaria Consortium / VCWG  2. MORU  3. Mahidol / MORU  4. Health GeoLab Collaborative |
| 14:55 | 00:05 | Conclude groupwork activity | Session Chair / Facilitators |  |
| 15:00 | 00:10 | From data to elimination:  finalize research priority and training needs | Prof Richard Maude | MORU / SRWG |
| 15:10 | 00:05 | Closing | Dr Rattanaxay Phetsouvanh | MoH Lao PDR / SRWG |
