# Supplementary material for: Gaps in research and capacity development for malaria surveillance and response in the Asia–Pacific: meeting report
Source: Malar J. 2023 Mar 10;22:91. doi: 10.1186/s12936-023-04459-9 (PMC10000341; doi:10.1186/s12936-023-04459-9)
Supplement: Supplementary file 2 — Additional file 2: List of attending country partner National Malaria Control Programmes and partner institutions. [file 12936_2023_4459_MOESM2_ESM.docx]

**Additional file 2.**

**List of attending country partner National Malaria Control Programmes and partner institutions**

| **Organization Name** | **Type of Organization** | **Country** |
| --- | --- | --- |
| Aga Khan University | Academic Institution | Pakistan |
| Kasetsart University | Academic Institution | Thailand |
| Universiti Malaysia Sarawak | Academic Institution | Malaysia |
| University of Oslo | Academic Institution | Norway |
| Australian National University | Academic Institution | Australia |
| James Cook University | Academic Institution | Australia |
| Menzies School of Health Research | Academic Institution | Australia |
| Jiangsu Institute of Parasitic Diseases | Academic Institution | China |
| Burnet Institute | Academic / Research Institution | Australia, Myanmar |
| Mahidol Oxford Tropical Medicine Research Unit | Academic / Research Institution | Bangladesh, Myanmar, South Africa, Thailand, United Kingdom |
| National Center for Vector Borne Disease Control (NVBDCP) | Government Agency | India |
| Ministry of Health | Government Agency | India |
| Anti Malaria Campaign | Government Agency | Sri Lanka |
| Australian Defence Force Malaria and Infectious Disease Institute (ADFMIDI) | Government Agency | Australia |
| Directorate General of Health Services | Government Agency | Bangladesh |
| Ministry of Health | Government Agency | Bhutan |
| Vector Borne Disease Control Program, Ministry of Health | Government Agency | Bhutan |
| National Center for Parasitology Entomology and Malaria Control | Government Agency | Cambodia |
| Center of Malariology Parasitology and Entomology, Department of Communicable Disease Control, Ministry of Health | Government Agency | Lao PDR |
| Ministry of Health | Government Agency | Malaysia |
| Directorate of Malaria Control | Government Agency | Pakistan |
| Ministry of National Health Services, Regulations & Coordination | Government Agency | Pakistan |
| National Department of Health | Government Agency | Papua New Guinea |
| Department of Health | Government Agency | Philippines |
| National Malaria Control and Elimination Programme | Government Agency | Philippines |
| Ministry of Health and Medical Services | Government Agency | Solomon Islands |
| National Vector Borne Disease Control Program | Government Agency | Solomon Islands |
| Division of Vector Borne Diseases Control | Government Agency | Thailand |
| National Institute of Malariology, Parasitology and Entomology | Government Agency | Vietnam |
| USAID/PMI | Government Agency | Thailand |
| Shoklo Malaria Research Unit | Non-government Organization / Research Institution | Thailand |
| Malaria Free Mekong, Regional CSO Platform – Greater Mekong Subregion | Non-government Organization | Australia |
| BRAC | Non-government Organization | Bangladesh |
| International Centre for Diarrhoeal Disease Research, Bangladesh (iccddr, b) | Non-government Organization | Bangladesh |
| Clinton Health Access Initiative | Non-government Organization | Cambodia, Lao PDR |
| University of Research, Co., LLC. | Non-government Organization | Cambodia, United States |
| Advance Health Care Foundation of India | Non-government Organization | India |
| Health Poverty Action | Non-government Organization | Myanmar |
| Myanmar Council of Churches | Non-government Organization | Myanmar |
| Myanmar Health and Development Consortium (MHDC) | Non-government Organization | Myanmar |
| Medical Action Myanmar | Non-government Organization | Myanmar |
| PATH | Non-government Organization | Myanmar, Vietnam |
| APMEN Vector Control Working Group | Non-government Organization | Thailand |
| APMEN Vivax Working Group | Non-government Organization | Nepal, Thailand |
| Indus Hospital and Health Network | Non-government Organization | Pakistan |
| Rotarians Against Malaria | Non-government Organization | Papua New Guinea |
| ACTMalaria Foundation, Inc. | Non-government Organization | Philippines |
| Asia Pacific Leaders Malaria Alliance | Non-government Organization | Singapore |
| Malaria Consortium | Non-government Organization | Thailand |
| World Vision Thailand | Non-government Organization | Thailand |
| Raks Thai Foundation | Non-government Organization | Thailand |
| Medicines for Malaria Venture | Non-government Organization | Thailand, United Kingdom |
| Supporting Community Development Initiatives | Non-government Organization | Vietnam |
| Innovative Vector Control Consortium | Private Sector | India |
| Abt Associates | Private Sector | Papua New Guinea |
| Health GeoLab Collaborative | Private Sector | Philippines |
| Pilipinas Shell Foundation, Inc (PSFI) | Private Sector | Philippines |
| University of California San Francisco | Research Institution | Lao PDR |
| Tribhuvan University | Research Institution | Nepal |
| Research Institute for Tropical Medicine | Research Institution | Philippines |
| Mahidol Vivax Research Unit, Faculty of Tropical Medicine, Mahidol University | Research Institution | Thailand |
| RTI International | Research Institution | United States |
| World Health Organization | UN Agency | Cambodia, Lao PDR, Papua New Guinea, Switzerland, Thailand, United Kingdom, United States |
| UNICEF | UN Agency | Indonesia |
| World Health Organization / Regional Office for the Western Pacific | UN Agency | Philippines |
