# Supplementary material for: Gaps in research and capacity development for malaria surveillance and response in the Asia–Pacific: meeting report
Source: Malar J. 2023 Mar 10;22:91. doi: 10.1186/s12936-023-04459-9 (PMC10000341; doi:10.1186/s12936-023-04459-9)
Supplement: Supplementary file 3 — Additional file 3: Breakout groups and identified research priorities on malaria surveillance in the region. [file 12936_2023_4459_MOESM3_ESM.docx]

**Additional file 3.**

**Breakout groups and identified research priorities on malaria surveillance in the region**

| **Subgroup** | **Country partners / Partner institutions** | **Research topics ranked by priority (voted in-group)** |
| --- | --- | --- |
| **Greater Mekong Subregion +** | **Cambodia, China (JIPD), Lao PDR, Thailand** | 1. Assessment of the sensitivity and specificity of the surveillance, monitoring and evaluation system  2. Integration of malaria surveillance with the general health system  3. Monitoring malaria among mobile and migrant populations  4. The minimum package of surveillance and monitoring and evaluation capacity for sustaining malaria free status |
| **South Asia** | **Bangladesh, Bhutan, Pakistan** | 1. Cross-border sero-surveillance  2. Geno-typing of malaria cases  3. Approaches for mobile and migrant population towards malaria elimination  4. Outdoor vector control measures  5. Ecology of a mosquito species (larval habitats) and its behaviour (host biting preferences) in high burden area  6. Gaps identification in malaria Surveillance specially in remote areas with low coverage of interventions  7. Study the effect of 14days PQ on radical cure  8. Uniform surveillance system for data recording and reporting  9. National survey for vector bionomics  10. National survey for asymptomatic malaria |
| **Melanesia and Malay Archipelago** | **Malaysia, Papua New Guinea, Philippines, Solomon Islands** | 1. Improved traps for mosquito surveillance (that are efficient as human landing catch) at catching Anopheles mosquitoes  2. Effective vector control methods for Knowlesi malaria  3. Improved community-based surveillance for malaria infection  4. Evaluation national malaria elimination surveillance strategy and tools  5. Research to support ongoing surveillance system |
| **Non-National Malaria Programmes** | **Non-National Malaria Programs/Partner institutions** | 1. Improving sharing of surveillance data across borders  2. Why does a particular group have persistent malaria?  3. How effective are different models for integrating malaria surveillance (e.g. surveillance system)  4. Best means to stop P.vivax relapses with 8-Aminoquinolines  5. What is the impact for COVID 19 on malaria reduction? Will it have the same impact in different countries in the region? |
| **Non-National Malaria Programmes** | **Non-National Malaria Programs/Partner institutions** | 1. What strategies for malaria elimination are best targeted for reaching and addressing malaria transmission among migrant/forest going populations?  2. What surveillance strategies are most cost effective and how do we maximize our limited resources for surveillance?  3. Operational research into how surveillance systems and data are used and where they can be improved?  4. Identifying bottlenecks to patient follow-up, especially among mobile populations  5. How can surveillance give Program Manager a better understanding of the heterogeneity of the malaria transmission for improved decision-making?  6. Factors need to consider to roll out Malaria vaccine in endemic areas  7. How can we effectively target vivax malaria? |
| **Non-National Malaria Programmes** | **Non-National Malaria Programs/Partner institutions** | 1. Population movement and control of malaria  2. Access to care for hard to reach populations  3. Effectiveness of data sharing platform for surveillance data and the importance in cross border collaboration  4. Supporting community engagement and ownership of surveillance and response at frontline  5. Adherence to radical cure tools for vivax malaria  6. Identification/monitoring of malaria drug resistance  7. How can surveillance in the private sector be integrated and not be resource intensive for private providers?  8. Ensuring rights-based approaches to surveillance and response  9. Effective/safe use of primaquine for vivax malaria  10. Diagnosis of vivax malaria  11. Determine optimal platform and information structure for integrated entomology & Epidemiological data interrogation and response formulation  12. How to identify areas/populations that present a risk for malaria resurgence?  13. Discover genetic markers for new antimalarials, such as Pyramax  14. How can serology support surveillance?  15. Ensuring adherence to P.vivax radical cure among mobile populations/hard to reach groups  16. How important is it to assess the different effectiveness of longer versus shorter 8-AQ treatments?  17. What do medical entomologists need, and which universities are providing basic degrees that can produce the next generation of medical entomologists  18. Are 8-aminoquinolines efficacious if they are provided after several weeks of blood-stage? |

**Research Questions ranked by Country Partner votes**

| **Research Question** | **Country partner votes** | **Partner Institutions votes** |
| --- | --- | --- |
| What strategies for malaria elimination are best targeted for reaching and addressing malaria transmission among migrant/forest going populations? | 6 | 13 |
| What surveillance strategies are most cost effective and how do we maximize our limited resources for surveillance? | 5 | 13 |
| How to integrate malaria surveillance with the broader health system | 5 | 8 |
| How to do cross border malaria surveillance | 4 | 3 |
| The minimum package of surveillance and monitoring and evaluation capacity for sustaining malaria free status | 3 | 7 |
| Outdoor vector control measures | 3 | 6 |
| Approaches to malaria elimination for mobile and migrant populations | 3 | 4 |
| Improved community-based surveillance for malaria infections and how best to do that | 3 | 3 |
| How to improve sharing of surveillance data across borders | 3 | 3 |
| How can surveillance give Program Manager a better understanding of the heterogeneity of the malaria transmission for improved decision-making? | 3 | 2 |
| How to monitor malaria among mobile populations, including mobile and immigrants? | 3 | 2 |
| Operational research into how surveillance systems and data are used and where they can be improved | 2 | 8 |
| Determine optimal platform and information structure for integrating entomology & epidemiological data interrogation and response formulation | 2 | 4 |
| Supporting community engagement and ownership of surveillance and response at the frontline | 2 | 2 |
| Access to care for hard to reach populations | 2 | 2 |
| Mobile and migrant movement mapping in elimination districts | 2 | 2 |
| Genotyping of malaria cases | 2 | 1 |
| How to identify areas/populations that present a risk for malaria resurgence? | 1 | 9 |
| Assessment of the sensitivity and specificity of the surveillance, monitoring and evaluation system | 1 | 6 |
| Best regimen for radical cure of P. vivax (duration, timing, efficacy, safety, ensuring adherence) | 1 | 4 |
| Ecology of a mosquito species (larval habitats) and its behaviour (host biting preferences) in high burden area | 1 | 3 |
| Operational research on routinely collected data | 1 | 3 |
| Identifying bottlenecks to patient follow-up, especially among mobile populations | 1 | 1 |
| Effectiveness of data sharing platforms for surveillance data | N/A | 4 |
| Why does a particular group/location have persistent malaria? | N/A | 4 |
| Population movement and control of malaria | N/A | 3 |
| What are the effective vector control methods for P. knowlesi Malaria? | N/A | 3 |
| Ensuring rights-based approaches to surveillance and response | N/A | 2 |
| Identification/monitoring of malaria drug resistance | N/A | 2 |
| How can surveillance in the private sector be integrated and not be resource intensive for private providers? | N/A | 2 |
| Improved traps for mosquito surveillance (that are as efficient as Human Landing Catch) at catching Anopheles mosquitoes | N/A | 2 |
